# Ultrasound diagnosis of three different types of carpal tunnel syndrome associated with persistent median artery: A case report and literature review

**DOI:** 10.1097/MD.0000000000047643

**Published:** 2026-02-28

**Authors:** Wenjiao Xu, Xin Jin, Baomin Liu

**Affiliations:** a Department of Ultrasound, Xi’an International Medical Center Hospital, Xi’an, Shaanxi, China.

**Keywords:** carpal tunnel syndrome, different types, persistent median artery, ultrasound diagnosis

## Abstract

**Rationale:**

Carpal tunnel syndrome (CTS) is the most common neuropathy worldwide. Although multiple factors contribute to CTS, persistent median artery (PMA)-related CTS is relatively uncommon. To date, no literature has reported the ultrasonic features of different types of PMA-induced CTS.

**Patient concerns:**

We report the clinical manifestations and ultrasonographic features of 3 patients with different types of CTS associated with PMA and review relevant literature.

**Diagnoses:**

All 3 cases were diagnosed with PMA combined with CTS, specifically categorized as CTS with PMA thrombosis, CTS with PMA and median nerve bifidity, and CTS with PMA and high bifurcation of the median nerve.

**Interventions:**

Different treatment measures were administered based on ultrasound diagnosis and clinical presentation, all with good prognoses.

**Lessons:**

Ultrasound enables precise and rapid identification of various PMA-related types of CTS, effectively guiding clinicians in making the most patient-friendly treatment decisions.

## 1. Introduction

**Figure 1.**
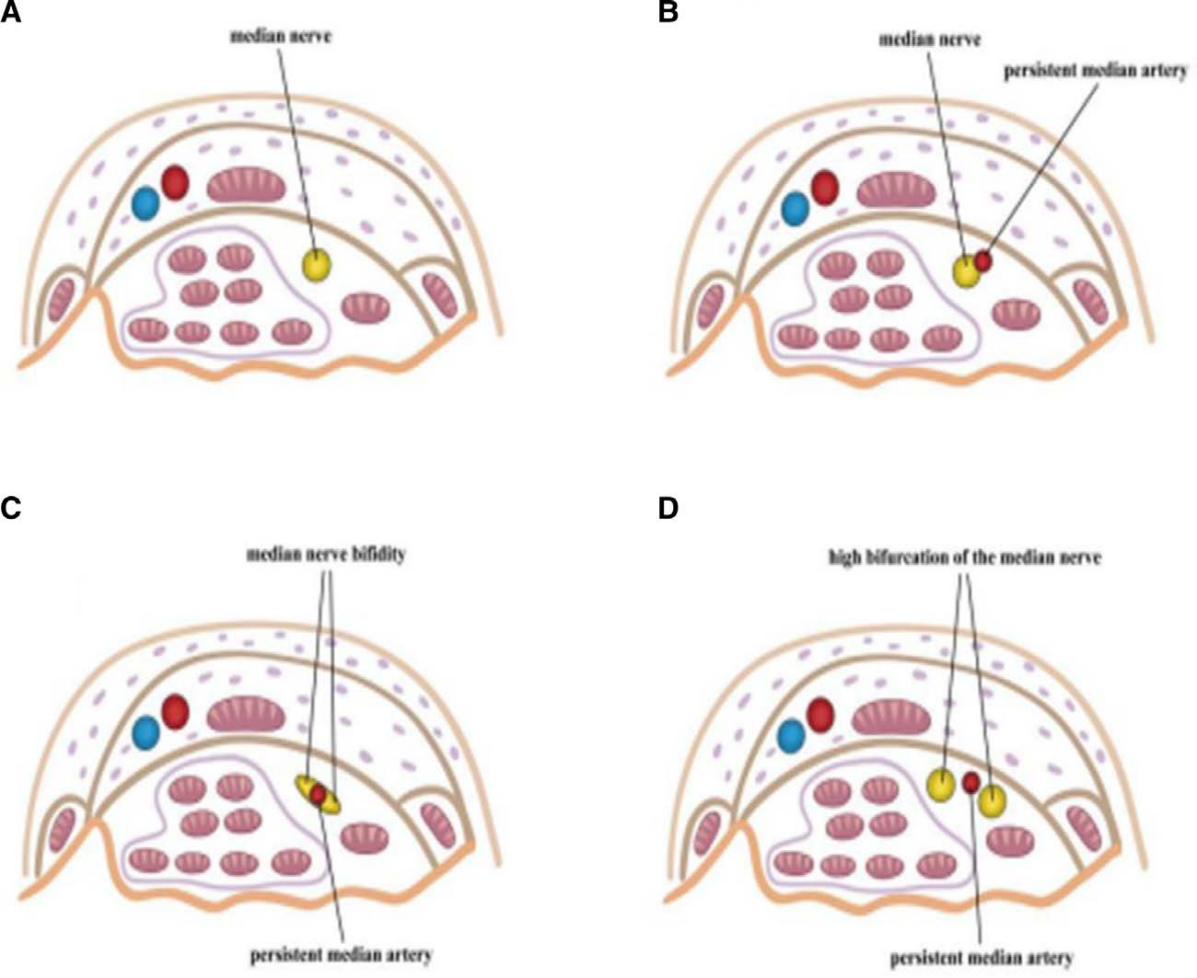
Schematic diagrams of 3 types of CTS related to PMA: the median nerve in its normal course (A), CTS with PMA thrombosis (B), CTS with PMA and median nerve bifurcation (C), and CTS with PMA and high bifurcation of the median nerve (D). CTS = carpal tunnel syndrome, PMA = persistent median artery.

Carpal tunnel syndrome (CTS) is a common peripheral entrapment neuropathy of the upper limb.^[1]^ Compression or injury of the median nerve, as it passes through the limited space of the osseofibrous carpal tunnel in the wrist causes CTS, leads to hand pain, numbness, and impaired function, affecting approximately 5% of the population.^[2]^ Its risk factors include age, sex, pregnancy, morbid obesity, repetitive wrist motions, diabetes, rheumatoid inflammation, genetic predisposition, anatomical variations, and the persistence of the median artery.^[3–5]^ Among these, CTS caused by a persistent median artery (PMA) is relatively rare, with a very low incidence. The median artery accompanies the median nerve in the forearm during embryonic development, supplying blood to the forearm and hand. With the development of the radial and ulnar arteries, the median artery typically atrophies and disappears. If the median artery persists, it is termed PMA. This study aims to summarize the ultrasonic characteristics of 3 types of CTS caused by PMA (Fig. 1), which are CTS with PMA thrombosis, CTS with PMA and median nerve bifidity, and CTS with PMA and high bifurcation of the median nerve in order to enhance the understanding of ultrasound physicians about this disease, reduce misdiagnosis rates, and provide a detailed basis for clinical treatment.

## 2. Patients and methods

We collected ultrasound images and results from 3 patients diagnosed with PMA combined with CTS by ultrasound at our hospital between January 2024 and May 2025. Ultrasound examinations were performed using a GE LOGIQ E10 system (USA) with an L3–12 linear array transducer. Due to the relative rarity of this condition, we reviewed and summarized relevant literature. This is a descriptive case series study aiming to characterize the clinical features and outcomes of patients with CTS. The primary objective is to provide a detailed account of individual cases rather than to draw statistical inferences. This study was approved by the ethics committee of Xi’an International Medical Center Hospital, with the approval number 202532. All participants provided informed consent.

## 3. Case reports

### 3.1. Case 1

**Figure 2.**
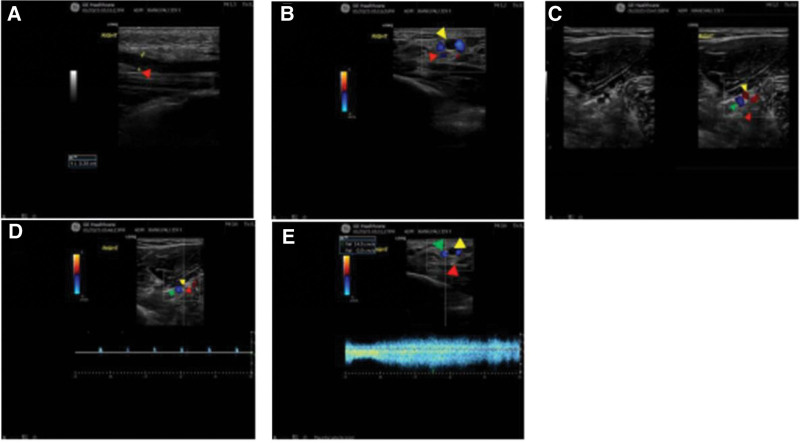
Anterior segment of median nerve (red arrow) shows permanent presence of median artery (yellow arrow) and vein (green arrow) (C), with patent lumen of artery and vein (D–E), passing through thickened and edematous wrist nerve (A), with thrombus filling in paratherm artery (B) (case 1).

A 40-year-old male patient came to our pain department 2 months ago due to right wrist pain, swelling, and occasional numbness of the thumb and index finger at night for 3 months. Physical examination revealed swelling of the soft tissues on the volar aspect of the right wrist, positive Tinel’s sign, and positive Phalen’s test over the right wrist. Ultrasound examination showed a continuous course of the right median nerve, with significant thickening and edema at the wrist level (Fig. 2A). The cross sectional diameter at the inlet was approximately 2.9 mm, area approximately 0.11 cm^2^; at the carpal tunnel ligament level, diameter was approximately 2.5 mm; distal to the outlet, diameter was approximately 3.3 mm, area approximately 0.15 cm^2^. Three tubular echoes were observed accompanying the median nerve (Fig. 2C), disappearing near the elbow. Near the wrist, 2 of these were anechoic tubular structures with a diameter of approximately 1.0 mm, filled with color flow signals; pulsed wave (PW) Doppler-recorded venous flow spectra in both, with a mean velocity of 14.5 cm/s (Fig. 2E). The middle tubular anechoic structure had a diameter of approximately 3.1 mm, filled with hypoechoic material, and showed no significant flow signal internally (Fig. 2B). Near the elbow, a low-velocity arterial flow spectrum could be recorded within its lumen (Fig. 2E). Ultrasound diagnosis: the median nerve of the right wrist is thickened and swollen, suggesting CTS, a tubular echo beside the median nerve of the right wrist, with one of the tubular echoes filled with low echogenicity, suggesting PMA and vein with thrombosis of the median artery. Clinical diagnosis: right CTS with thrombosis of a PMA. The pain management team prescribed rest, wrist immobilization, mecobalamin nerve nutrition therapy, and rivaroxaban anticoagulation treatment. After 1 month of follow-up, the patient reported significant.

### 3.2. Case 2

A 56-year-old male patient came to our pain medicine department 3 months ago because of numbness in his right thumb, index finger, and middle finger for half a year.

**Figure 3.**
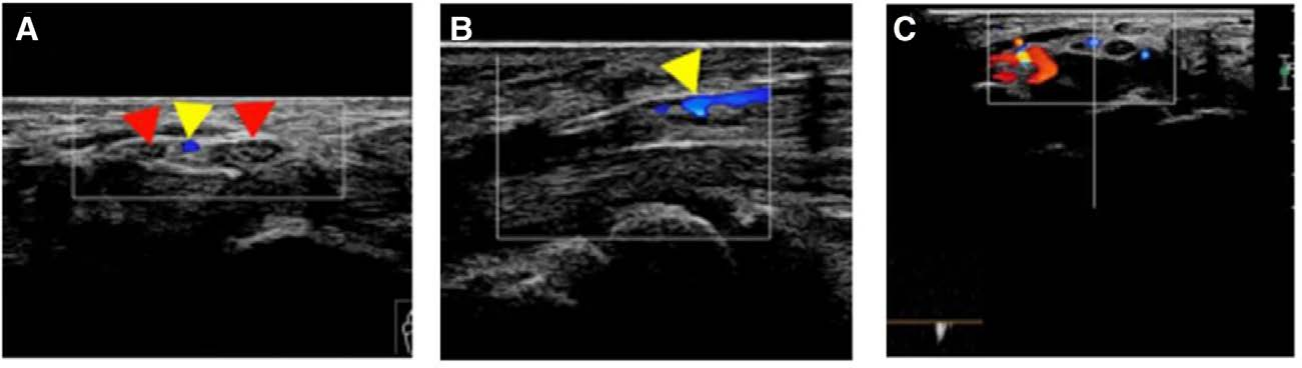
The median radial nerve (red arrow) is divided into 2 bundles by the permanent median artery (yellow arrow). The 2 bundles of nerves have their own outer membranes (A), and blood flow can be seen between the 2 bundles of nerves (B–C) (case 2).

Physical examination revealed weakened grip strength in the right hand compared with the contralateral side, positive Tinel’s sign, and positive Phalen’s test over the right wrist. Ultrasound examination showed a continuous course of the right median nerve. A tubular anechoic structure approximately 0.19 cm in diameter was seen within the midportion of the nerve, dividing the nerve bundles into left and right bundles (Fig. 3A). Both bundles had their own epineurium. Color Doppler showed flow signals within the tubular anechoic structure (Fig. 3B). PW: recorded arterial spectrum, Vmax 35 cm/s, RI 0.54 (Fig. 3C). The diameters of the 2 nerve bundles within the carpal tunnel were approximately 1.9 mm (radial bundle) and 1.2 mm (ulnar bundle), respectively. Ultrasound diagnosis: the thickening of the median nerve on the right wrist suggests possible CST, 2 bundles of the median nerve at the right carpal tunnel, each with its own perineurium, indicate a high-level bifurcation of the median nerve, and a tubular echo and blood filling between the 2 bundles of the median nerve on the right wrist suggest a PMA. Clinical diagnosis: right CTS with PMA and median nerve bifidity. Due to the severity of symptoms, the patient consented to ligation of the PMA after discussion. At the 6-month follow-up, the artery was occluded and the patient’s symptoms were significantly relieved.

### 3.3. Case 3

**Figure 4.**
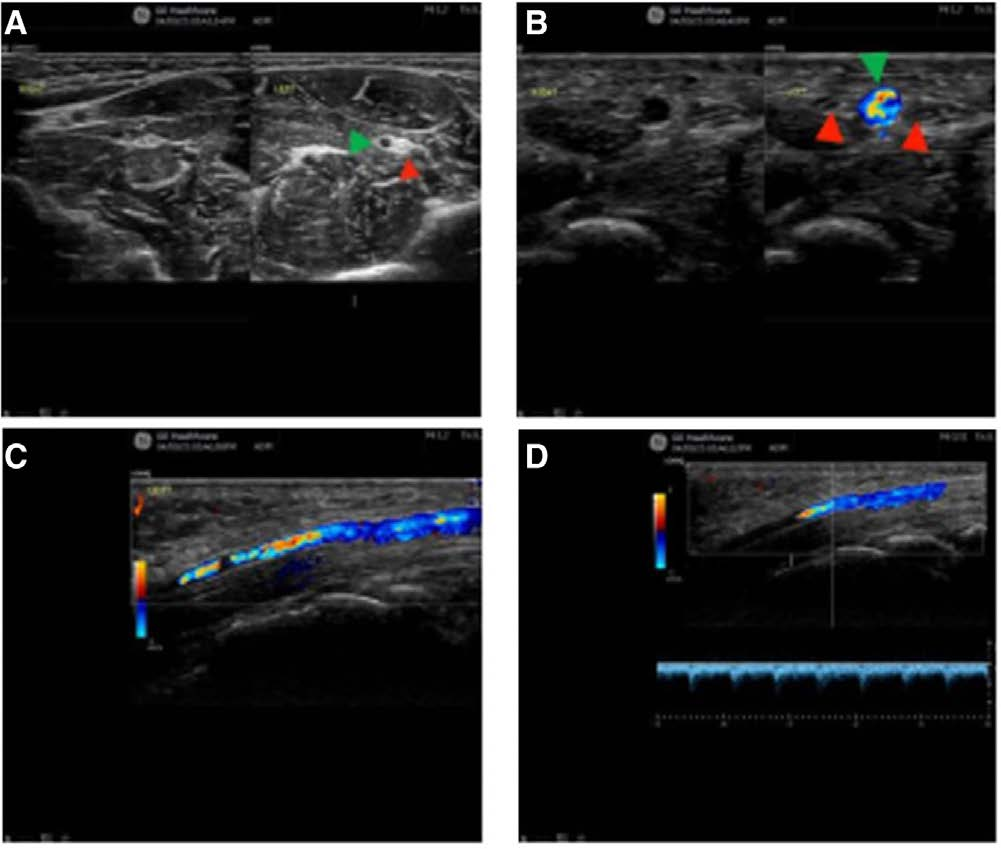
Tubular anechoic area (green arrow) visible within the median nerve in the forearm segment (red arrow) (A), with blood flow filling visible within the tubular anechoic area (green arrow) (B–D) (case 3).

A 49-year-old male patient came to our pain department 1 month ago because of numbness, swelling, and pain in his right hand for 1 month. The symptoms worsened at night and could be relieved by activity or hand tremor. Physical examination revealed positive Phalen’s test and Tinel’s sign. Ultrasound examination showed a continuous course of the right median nerve. A tubular anechoic structure was visible within the nerve, surrounded by nerve bundles (Fig. 4A). Color Doppler showed color flow filling the anechoic structure (Fig. 4B–C). PW recorded an arterial spectrum, Vmax: 16 cm/s (Fig. 4D). The cross sectional areas of the nerve were approximately inlet 0.18 cm^2^, carpal tunnel ligament level 0.11 cm^2^, and outlet 0.16 cm^2^. Ultrasound diagnosis: the median nerve in the right wrist is thickened, suggesting CTS, at the right carpal tunnel, there is a bundle of the median nerve with tubular echogenicity within the nerve and blood flow filling. Clinical diagnosis: right CTS with PMA and high bifurcation of the median nerve. Due to mild symptoms, conservative treatment was taken after communication with the patient. The patient was instructed to rest and given methylcobalamin for neurotrophic treatment. After 1 month of follow-up, the patient reported no obvious numbness or pain symptoms and the treatment effect was good.

## 4. Literature review and discussion

The incidence of CTS is as high as 4%. With the continuous development of musculoskeletal ultrasound and electromyography, the diagnostic rate for CTS is increasing. There are many risk factors for CTS, among which CTS associated with PMA is relatively rare. PMA is classified into the forearm type (not reaching the wrist) and the palmar type (extending to the wrist through the carpal tunnel), the latter being a potential cause of CTS.^[6–8]^ This article summarized the clinical and ultrasonic features of 3 cases associated with PMA (Table 1) and summarized the characteristics of CTS combined with PMA in these 3 cases: CTS with PMA thrombosis, CTS with PMA and median nerve bifidity, and CTS with PMA and high bifurcation of the median nerve. Median nerve bifidity refers to the nerve splitting into 2 bundles within a common epineurium at the carpal tunnel, whereas high bifurcation of the median nerve means the nerve is completely divided into 2 distinct bundles, each with its own epineurium. When PMA develops thrombosis, the vessel diameter rapidly increases, raising pressure within the carpal tunnel and causing acute compression of the median nerve. High bifurcation and bifidity of the median nerve increase its cross sectional area; combined with the presence of PMA, this leads to CTS. Relationship between PMA thrombosis and CTS: direct compression: when thrombi form in the PMA, the enlarged vessel volume directly compresses the median nerve within the carpal tunnel, causing nerve dysfunction manifested as hand numbness and tingling sensations. Increased tunnel pressure: thrombosis elevates intravascular pressure, further increasing overall pressure within the carpal tunnel and exacerbating nerve ischemia and dysfunction. Relationship between PMA branching and CTS: anatomical variations: the PMA is often associated with median nerve bifurcation or branching patterns, such as when the median nerve divides into 2 bundles enveloping the artery or undergoes high-level bifurcation. These anatomical variations alter the relative positioning between nerves and blood vessels, significantly increasing neural compression risks. Dynamic compression: during wrist movement, friction and traction between branched nerves and the PMA intensify, potentially causing nerve injury and inflammatory responses that worsen CTS symptoms. Pathophysiological mechanisms include ischemia and compression: thrombosis induces vascular occlusion leading to nerve ischemia, while anatomical variations create a vicious cycle where compressed nerves exacerbate compression. Inflammatory response: thrombus-induced nerve compression triggers localized inflammation releasing mediators that damage nerves and surrounding tissues. Neurological dysfunction: chronic compression and ischemia slow nerve conduction velocity, impair sensory and motor functions, ultimately manifesting as CTS symptoms. This mechanism underscores the critical role of PMA variations and thrombosis in CTS pathogenesis, providing crucial diagnostic and therapeutic guidance. Different treatment plans were adopted based on ultrasound diagnosis and clinical symptoms, and symptoms were relieved after various treatments. This demonstrates the crucial role of ultrasound in the accurate diagnosis and management of this condition.

**Table 1:** The clinical and ultrasonic features of 3 cases associated with PMA.

Project	Case 1	Case 2	Case 3
Symptom	Arm pain and swelling, occasional numbness of thumb and index finger at night	Numbness in thumb, index and middle fingers of right hand	Numbness and swelling pain in the right hand
Duration	3 mo	6 mo	1 mo
Diagnose	Right CTS with PMA and vein, with thrombosis of median artery	Right CTS with permanent high bifurcation of median artery and median nerve	Right CTS with permanent midarterial and median nerve fissures
Ultrasound image features	The right median nerve was thickened and edematous with tubular echoes adjacent to it, and the tubular echoes were filled with hypoechoic areas	The right median nerve is seen in 2 bundles with their own neural outer membrane, and a tubular echo between the 2 bundles with blood flow filling within the tubular echo	The right median nerve was thickened with tubular echoes and blood flow filling within the nerve bundles
Therapeutic method	Rest, immobilization of the wrist, mecobalamin for nerve nutrition, rivaroxaban for anticoagulation	The middle artery was ligated surgically and permanently	Rest, mecobalamin nourishes nerves
Follow-up time after treatment	1 mo	6 mo	1 mo
Follow-up symptoms after treatment	Pain and numbness were significantly improved	Symptoms are significantly relieved, occasionally numb	No obvious numbness or pain

CTS = carpal tunnel syndrome, PMA = persistent median artery.

We systematically searched relevant literature in databases. From 2015 to 2025, a total of 10 relevant articles were retrieved. One article reported an incidence of PMA of approximately 8.3% among CTS patients.^[9]^ Four articles reported cases of acute CTS caused by PMA thrombosis,^[10]^ but these were confirmed by magnetic resonance imaging (MRI) or surgery and did not summarize the ultrasonographic features. Two articles were case reports on PMA thrombosis combined with median nerve bifurcation.^[11]^ The remaining 3 articles primarily discussed the anatomy of PMA. Our study focuses on the specific ultrasound-based classification of CTS associated with PMA, which has not been reported previously. Ultrasound, MRI, and surgical diagnosis have distinct advantages in confirming PMA or anatomical variations of the median nerve with CTS: ultrasound: the primary screening tool, offering dynamic real time imaging to precisely display PMA blood flow, median nerve bifurcation (low/high), and nerve compression patterns (e.g., notch sign, nerve swelling). Quantitative diagnosis: direct measurement of key indicators. Etiological identification: the only imaging method capable of simultaneously detecting PMA, nerve variations, and secondary lesions (e.g., vascular calcification, tenosynovitis). Noninvasive convenience is cost-effective and radiation-free, and can guide minimally invasive treatments. Limitations include reliance on operator experience and limited resolution of subtle nerve abnormalities (e.g., fibrosis). MRI serves as a supplementary tool for complex cases, with advantages including high soft tissue contrast to clearly visualize internal nerve structures (edema, fibrosis) and surrounding soft tissue lesions (tumors, synovitis). Panoramic imaging evaluates overall carpal tunnel anatomy, particularly suitable for high nerve bifurcation or postoperative assessment. Limitations are inability to observe real time dynamic nerve mobility, high cost, time-consuming procedures, and potential interference from intrabody metal implants. Surgical diagnosis: the gold standard but not the preferred approach, with advantages including direct decompression (e.g., transverse wrist ligament incision) and simultaneous handling of PMA or nerve variations, ensures definitive therapeutic outcomes. The limitation is invasive nature of surgery, only applicable for cases unresponsive to conservative treatment or those with muscle atrophy, and cannot replace preoperative precise evaluation. MRI can provide an overall view of the neural structure. However, ultrasound is significantly superior to MRI in displaying the fine neural structures. For example, when differentiating between median nerve bifurcation and median nerve cleft, the key is to observe whether the palmar median artery divides the median nerve into 2 separate fascicles, that is, whether they have their own epineurium. If they have their own epineurium, it is considered median nerve bifurcation; otherwise, it is a median nerve cleft. Superficial ultrasound plays an irreplaceable role in observing the epineurium. When differentiating whether the PMA is accompanied by a thrombus, ultrasound can detect blood flow signals within the artery to determine if a thrombus has formed. Therefore, ultrasound is also superior to MRI in observing blood flow signals. As a result, ultrasound has unique advantages in the differentiation of different subtypes.

This study has several limitations. First, the relatively small sample size may affect the generalizability of the results. Second, although the cases were followed up, the follow-up period was short and lacked long-term monitoring. Despite these limitations, the findings still provide important references for differentiating PMA combined with CTS through ultrasound. Future studies could improve these shortcomings by expanding the sample size.

## 5. Conclusion

Ultrasound enables precise and rapid identification of various PMA-related types of CTS, effectively guiding clinicians in making the most patient-friendly treatment decisions.

## Acknowledgments

The authors thank all of the patients who participated in the study.

## Author contributions

**Conceptualization:** Wenjiao Xu, Xin Jin, Baomin Liu.

**Data curation:** Wenjiao Xu, Baomin Liu.

**Formal analysis:** Wenjiao Xu.

**Resources:** Xin Jin.

**Supervision:** Xin Jin.

**Writing – original draft:** Wenjiao Xu.

**Writing – review & editing:** Wenjiao Xu, Baomin Liu.
